# How people affected by Chagas disease have struggled with their negligence: history, associative movement and World Chagas Disease Day

**DOI:** 10.1590/0074-02760220066

**Published:** 2022-07-13

**Authors:** Wilson Alves de Oliveira, Jordi Gómez i Prat, Pedro Albajar-Viñas, Cristina Carrazzone, Simone Petraglia Kropf, Aurore Dehousse, Ana Maria de Arruda Camargo, Mariella Anselmi, Maria Cristina Parada Barba, Isabel Claveria Guiu, Maria das Neves Dantas Silveira Barros, Maria da Glória Melo Cavalvanti, Cassandra Barros Correia, Silvia Marinho Martins, FINDECHAGAS Workgroup

**Affiliations:** 1 Universidade de Pernambuco, Ambulatório de Referência Estadual em Doença de Chagas, Casa de Chagas, Recife, PE, Brasil; 2 Associação Pernambucana de Portadores de Doença de Chagas, Recife, PE, Brasil; 3 International Federation of Associations of People Affected by Chagas, FINDECHAGAS Advisory Board, Campinas, SP, Brasil; 4 Unitat de Salut Internacional Vall d’Hebron-Drassanes, Programa de Salut Internacional del Institut Català de la Salut Equip de Salut Pública i Comunitària, Barcelona, Catalonia, Spain; 5 Asociación de Amigos de las Personas con la Enfermedad de Chagas, Barcelona, Catalonia, Spain; 6 World Health Organization, Department of Control of Neglected Tropical Diseases, Geneva, Switzerland; 7 Fundação Oswaldo Cruz-Fiocruz, Casa de Oswaldo Cruz, Departamento de Pesquisa em História das Ciências e da Saúde, Rio de Janeiro, RJ, Brasil; 8 Universidade de Campinas, Campinas, SP, Brasil; 9 Associação dos Portadores de Doença de Chagas de Campinas e Região, Campinas, SP, Brasil; 10 Centro Epidemiología Comunitaria y Medicina Tropical, Esmeralda, Ecuador; 11 Associazione Italiana di Lotta Alla Malattia de Chagas, Bergamo, Lombardia, Italy; 12 Asociación de Chagas de la Comunidad de Valencia, Valencia, Spain; 13 FINDECHAGAS Workgroup, Campinas, SP, Brasil

**Keywords:** Chagas disease, history, people affected, associative movement, world federation, World Chagas Disease Day

## Abstract

It is well documented that Chagas disease (CD) can pose a public health problem to countries. As one of the World Health Organization Neglected Tropical Diseases undoubtedly calls for comprehensive healthcare, transcending a restricted biomedical approach. After more than a century since their discovery, in 1909, people affected by CD are still frequently marginalised and/or neglected. The aim of this article is to tell the story of their activism, highlighting key historical experiences and successful initiatives, from 1909 to 2019. The first association was created in 1987, in the city of Recife, Brazil. So far, thirty associations have been reported on five continents. They were created as independent non-profit civil society organisations and run democratically by affected people. Among the common associations’ objectives, we notably find: increase the visibility of the affected; make their voice heard; build bridges between patients, health system professionals, public health officials, policy makers and the academic and scientific communities. The International Federation of Associations of People Affected by CD - FINDECHAGAS, created in 2010 with the input of the Americas, Europe and the Western Pacific, counts as one of the main responses to the globalisation of CD. Despite all the obstacles and difficulties encountered, the Federation has thrived, grown, and matured. As a result of this mobilisation along with the support of many national and international partners, in May 2019 the 72nd World Health Assembly decided to establish World Chagas Disease Day, on 14 April. The associative movement has increased the understanding of the challenges related to the disease and breaks the silence around Chagas disease, improving surveillance, and sustaining engagement towards the United Nations 2030 agenda.

**A disease of neglected people included in the first World Health Organisation list of Neglected Tropical Diseases**

People with *Trypanosoma cruzi* infection, or Chagas disease (CD), have been already diagnosed in forty four countries of five continents, but the low proportion of people diagnosed and treated - <10% - poses a great public health challenge.^1^ ^,^ ^2^ ^,^ ^3^ The World Health Organisation (WHO) estimates that between six and seven million individuals worldwide are currently infected with *T. cruzi*, in both endemic and non-endemic territories.

In the first half of the XXth century, Latin America received significant waves of migrants, especially from Japan and Europe, who mainly settled in rural areas and entered in contact with the trypanosome infection cycle. In the 1970s and 1980s, the urbanisation phenomenon led to a shift in the epidemiological profile of CD, placing the majority of the affected in urban centres and especially in poor, peripheral neighbourhoods. Moreover, the migratory movements at the end of the 20th century have significantly increased the spread of the disease outside Latin America and complexified the needed comprehensive healthcare for CD around the world.^4^

In 2005, the first World Health Organisation (WHO) list of Neglected Tropical Diseases (NTD), including CD, was published and the WHO Department of Control of NTDs, created,^5^ ^,^ ^6^ From the very beginning, the definition of this group of diseases is based on psychosocial rather than biomedical criteria.^7^

As a peculiar NTD, CD is a complex socio-economic, environmental health problem and its different interlinked dimensions justify the necessity of a multidimensional approach^8^ (Table I). All of that, together with the low visibility and little political power of the affected people, has underlined the need for them to gather together into civil organisations. Moreover it has enabled them to represent their interests, fight challenges surrounding CD - notably the barriers to healthcare or to CD control, or more simply, break the epidemiological silence around the disease.^2^

**TABLE I:** Multidimensional reality of Chagas disease with its complex socio-economic, environmental health dimensions

BIOMEDICAL ASPECTS	
1.1	Mainly a chronic and silent (asymptomatic/oligosymptomatic) disease affecting neglected and consequently silenced people facing different possible conditions: (i) infection carrier without clinical manifestations or indeterminate form (> 60% of cases); (ii) illness, with cardiac, digestive or other clinical manifestations; (iii); disease complications, such as vascular accident with frequent disability.
PERSONAL AND SOCIAL DETERMINANTS	
2.1	Personal psychological challenges, such as fear, shame or isolation, related to the disease itself or social environment.
2.2	Social challenges, such as stigma, exclusion, inequality, discrimination, barriers to access healthcare.
2.3	Economic inequalities related to diagnosis, treatment, and labour and social life conditions.
2.4	Limitations on social security and labour rights.
2.5	Gender inequalities related with social, psycho and cultural contexts.
PSYCHO-SOCIO-ANTHROPOLOGICAL CHALLENGES	
3.1	Women of childbearing age face the risk of congenital transmission , and are known to confront multiple and invisible psychological, familial, social, medical consequences.
3.2	Stereotypes on the affected population, seen as uneducated, unqualified, without agency (sociologically speaking, the ability of individuals to choose or to make independent and free decisions).
3.3	Frequent out of date and stereotyped concepts about the disease and the way to deal with it (present only in rural areas in Latin America, affecting exclusively poor populations, fatal disease without any available treatment).
INFORMATION AND COMMUNICATION COMPONENTS	
4.1	Unequal information and education, lack of communication about the disease, the rights of the affected ones.
4.2	Consequent general misinformation, low interest, lack of social demand and weak political commitment to solve problems related with CD.
RESEARCH AND DEVELOPMENT	
5.1	Insufficient scientific research and development related with prevention, detection and comprehensive care, including diagnosis, treatment, medicine presentations, social aspects, information, education and communication (IEC) tools, etc.

**The discovery of the disease and the first records of affected population’s voices**

At the turn of the XXth century, medical science experienced an increased optimism and confidence in the new knowledge acquired on infectious diseases, especially parasitic diseases transmitted by insect vectors. In a context marked by colonialism, institutions dedicated to research and education of tropical medicine were created in England and other European countries, making this topic a new specialty in its own right. Something similar also took place in Brazil. In response to the epidemics that ravaged the capital of the country (back then Rio de Janeiro), the Federal Serum Therapy Institute, also known as the Institute of Manguinhos, was created in 1900 to ensure the production of serums and vaccines. Its director, the young physician and researcher Oswaldo Cruz, had been trained at the Rio de Janeiro Faculty of Medicine, but also in the pioneer Institute Pasteur, in Paris. The new institution (which would be named after its director in 1908 and would give rise to the current Oswaldo Cruz Foundation) would soon become a respected research and teaching centre in microbiology and tropical medicine.^9^

Carlos Chagas discovered the disease that bears his name when he was working on the study and control of a malaria epidemic in Lassance, a small and poor city in the interior of the state of Minas Gerais (Brazil). It was celebrated as a milestone in Brazilian science since it was a triple discovery. On April 14th 1909, Carlos Chagas identified the first person affected by the disease. Before he had already identified its insect vector, known in Brazil as “barbeiro”, and its pathogen, a new species of trypanosome, named *T. cruzi* in honour of Oswaldo Cruz ^9^ (Fig. 1).

**Fig. 1:**
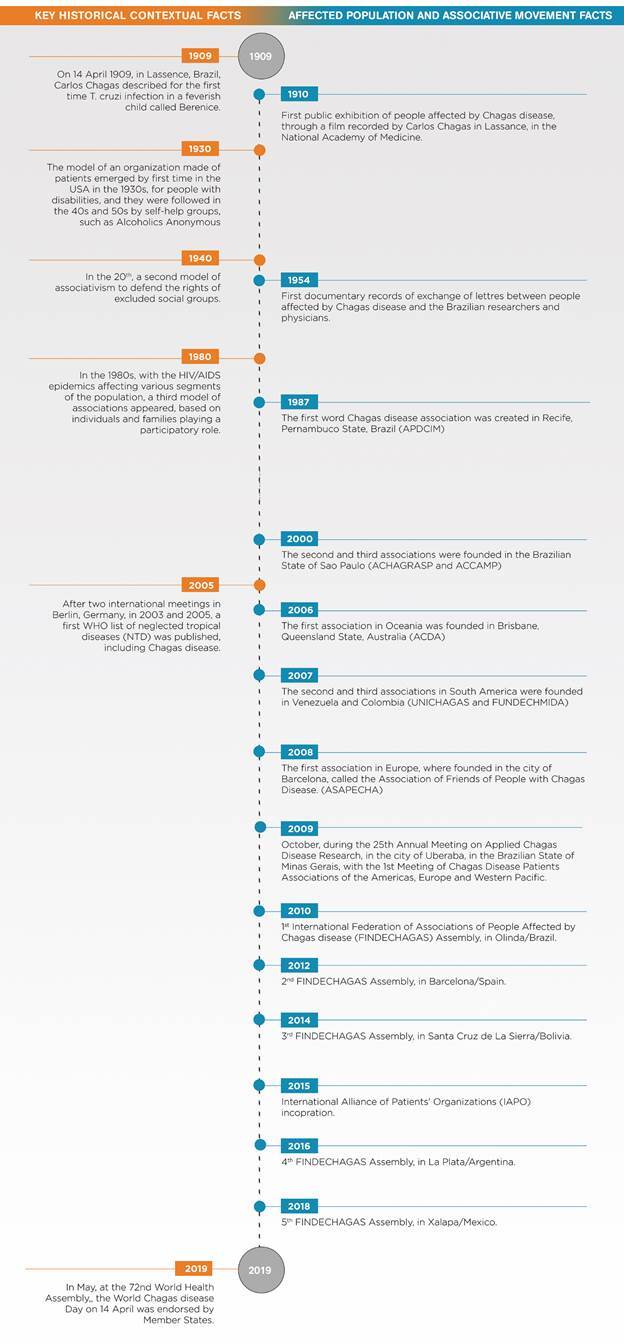
timeline 2009-2019. History of the affected population, associative movement, international federation and World Chagas disease Day.

Carlos Chagas always made a point of drawing attention to the importance of CD as both a medical and social problem that seriously affected the population of the interior of Brazil, also more at risk of other endemic diseases. On the occasion of his inauguration as a full member of the National Academy of Medicine of Brazil, in 1910, Chagas showed the faces of people who lived in miserable conditions by projecting a film he himself recorded in Lassance. Those images directly contradicted the “civilisation” that elites celebrated in the newly remodelled federal capital of the young republic. As a leader of the movement for rural sanitation in Brazil, he intensified his claim that the human American trypanosomiasis was directly related to the precarious living conditions of those populations. The poor housing conditions were evident and, in a vicious circle, contributed to further aggravate this precariousness (acting as key determinants of health). As director of the federal public health services, from 1919 onwards, he committed to creating health services and implementing necessary measures to serve these populations in every state of the country.^9^

Despite Chagas’ effort to put the social dimension of people affected by CD at the centre of his speeches and work, the people affected by the disease were not protagonists and were only made visible and heard through the voices or words of scientists. Furthermore, the absence of any records produced directly by these affected people, or at least their non-preservation in the historical archives, contributed a lot to this silencing. However, in some exceptional situations, the voices of these people were effectively present and were preserved, like in the following case. In 1943, the Oswaldo Cruz Institute appointed Emmanuel Dias, one of Carlos Chagas disciples, to lead the study of the disease in a field post in the city of Bambuí, state of Minas Gerais. The work developed by the Bambuí Centre was decisive to further knowledge on human American trypanosomiasis. Like Chagas, Dias played an active role in raising awareness on the medico-social aspects of the disease. Working directly in the endemic area, he received many letters from residents of the region, which were fortunately preserved in the Centre’s Collection, now under the custody of the Casa de Oswaldo Cruz - Oswaldo Cruz Foundation (Fiocruz) (see René Rachou Research Centre Fund/Advanced Research Post Section Emmanuel Dias, Department of Archives and Documentation, Casa de Oswaldo Cruz/Fiocruz).^9^ ^,^ ^10^

Many of those letters, written in a precarious Portuguese, reveal the scarce access to education in those regions and the real perception of the people of rural areas affected by CD. In general, they sought help from Dias for medical assistance, declaring that they had “heard’’ that the Bambuí Centre offered “remedies” for the illness. The letters also show that the piece of news on an insecticide able to fight the disease (in 1948 the BHC effectiveness against triatomine bugs was proved) was sometimes wrongly understood as a therapeutic resource and not as a mean of prevention. This is illustrated by the following section, from a letter of 1954 intended for Dias and written by Franscisco José da Silva, a resident of Claudio, a town in the interior of the state of Minas Gerais. He wrote: “(...) I thought it was not a dangerous disease, so I didn’t bother to treat it, but now I’m feeling my heart failing too much, it hits one blow and misses another, my legs are also a little swollen. For this reason I ask you to send me a prescription for the miraculous remedy, if necessary I will pay you the prescription (...)”. In response, Dias informed the man that, unfortunately, the aforementioned remedy did not exist, and made himself available for an examination at the Bambuí Centre. However, shortly thereafter, he received a letter from a relative informing him of Franscisco’s death.^9^

Sometimes, individuals affected by the disease actually request actions to fight against the insect vector. In 1957, a telegraph operator from Araxá (state of Minas Gerais), Joaquim Simões de Oliveira Filho, sent Dias a triatomine bug that had bitten his son. He complained about the lack of fight against the disease in his city and declared that he would help Dias in any necessary action to draw the attention of the authorities. In response, communicating the result of non-infection in the bug examination, Dias informed that he had sent his letter to the Minister of Health, as one more means of putting pressure on the health services to undertake the fight against the vectors of the disease in the region.^9^

The letters received by Dias from people affected by the disease were not many, or at least not many of them had been preserved. They are extremely valuable and representative documents of affected people’s experiences since they correspond to the first historical records of those at the forefront who communicated their anxieties and aspirations with their best available language. The presence of these voices in this article is a tribute to those who have dared speaking up about the concrete experience of their condition, which is still unfortunately neglected.^9^ Those are key for this study because they correspond to the roots of the organisations of people affected by CD. In the 1950’s they did not yet regroup in organisations to make themselves heard. In the years that followed, nevertheless, with little means they faced big challenges, originated a world associative movement and got the establishment of the World Chagas Disease Day, which gives the opportunity to all voices - past and present - to be heard at the international public level (Fig. 1).

**Patients associations. History of Chagas disease associative movement**

*The associative movement in the world* - Patients associations are voluntary groups of individuals that share certain biomedical and/or social characteristics. Patients organisations differ sometimes in their ultimate aim: some provide orientation to patients and their families in terms of treatment and quality of life improvement, others call for an active participation in the development and implementation of public health policies. These associations aim to provide a wide platform for affected individuals and their representatives open to the population at risk and even the general population.^11^ ^,^ ^12^

The first patients’ associations emerged in the 1930s, in the United States of America, for people with disabilities, and these were joined, in the 1940s and 1950s, by self-help groups such as Alcoholics Anonymous (AA). This model based on a collective identity among people sharing the same individual experiences and knowledge became popular worldwide. Although such organisations still exist, they have been criticised for failing to interact with other sectors of society. Later on, a second movement emerged that aimed to defend the rights of people excluded from society and this opposed a purely medical identity to the detriment of active patient participation.^12^ ^,^ ^13^

In the 1980s, a third model of associations appeared with HIV/AIDS beginning to affect various segments of the population. The affected individuals, their families and friends play an active role. More inclusive, this model better represents all three parts of the “treatment triad” of patient, family and health care team.^14^

*Chagas disease and the associations spread in the world* - CD associations took advantage of the history of the patients associations movements and the symbiosis of the different models. Citizenship progressively became a subject of collective debate, in which the organised civil society is given legitimacy in being an active social actor, interacting with all involved institutions and organisations, and even expressing an opinion on public health policy decision-making.

The first world CD association was created in the city of Recife, in the Brazilian state of Pernambuco, in 1987, almost eighty years after its discovery. The “Associação Pernambucana de Portadores de Doença de Chagas” (APDCIM) was created through the meeting of an innovative CD Outpatients Team and motivated patients from the University of Pernambuco’s Oswaldo Cruz Teaching Hospital - HUOC, under the slogan “A Commitment to Life”. It calls for an encompassing approach of CD, that addresses both its biomedical and social dimensions.^15^

However, it was only in 2000 that CD patients associations really started spreading. The “Associação dos chagásicos da Grande São Paulo” (ACHAGRASP), in the city of São Paulo, and the Associação dos Portadores de Doença de Chagas de Campinas e Região” (ACCAMP), in the municipality of Campinas, respectively the second and the third CD patients associations, were created. The meeting and collaboration between motivated patients and sensitive health professionals of the Service of Clinical Cardiology/Institute of Cardiology Dante Pazzanese and Group of Studies on Chagas disease (GEDoCh)/Unicamp, were fundamental to settle the objectives of both associations, including: fostering cooperation and mutual support and solidarity among patients with CD and their families, as well as promoting awareness raising, educational activities and defending civil rights.

All organisations outside Latin America were created by and with migrants from that territory to make their voice heard in their host countries. The first association of people affected by CD in Oceania, the Australian Chagas Disease Association, was founded by Latin American migrants in the city of Brisbane, Queensland state, Australia, in 2006, with the objective of working in a non-endemic country where the disease is mostly unkown and not only affects the poorest ones; fighting against indiference, desinformation, and discrimination towards people suffering due to CD. In 2008, the first association in Europe, “Asociación de Amigos de las Personas con la Enfermedad de Chagas” (ASAPECHA), was founded in the city of Barcelona by Latin American migrants together with members of the team of public and community health (espic) of the Unit of International Health Drassanes - Vall d’Hebron Hospital/Catalan Institute of Health, with the aim of helping infection carriers to organise among themselves and promote access to information and comprehensive care.

In 2012, the first association in North America, the “Asociación Mexicana de Personas Afectadas por la Enfermedad de Chagas” (AMEPACH), was founded in the city of Oaxaca de Juarez, state of Jalapa, Mexico. The association gathered affected individuals and researchers from up to 13 states of the country, with the aim of uniting patients and collaborators in the fight against delays in information, communication, diagnosis, treatment and care of people affected by the disease in the country, as well as public policies.

In 2021, in the city of Nagasaki, Japan, the first association in the Asian continent, Asociación Nipona de Afectados de Chagas (ANACHA), was constituted with the support of researchers from Nagasaki, Tokyo and even the Spanish Autonomous Community of Andalusia, who were working with CD in the country. The overarching statute’s objectives include collaborating on the design and implementation of research projects and disease control strategies with the health and social services (social, legal and economic assistance), academy and researchers, public and private organisations and institutions in Japan, but also in Latin America. The focused target population include the affected people and their families, migrant population at risk and health professionals. The principal work subjects include: information, education and communication (IEC), comprehensive healthcare, health promotion and research.

Thirty associations of people affected by CD have been reported on five continents: Asia, Europe, North America, Oceania and South America (Fig. 2). The list of the fourteen countries with CD associations include: Argentina, Australia, Bolivia, Brazil, Colombia, Ecuador, France, Italy, Japan, Mexico, Spain, Switzerland, the United States of America and Venezuela.

**Fig. 2:**
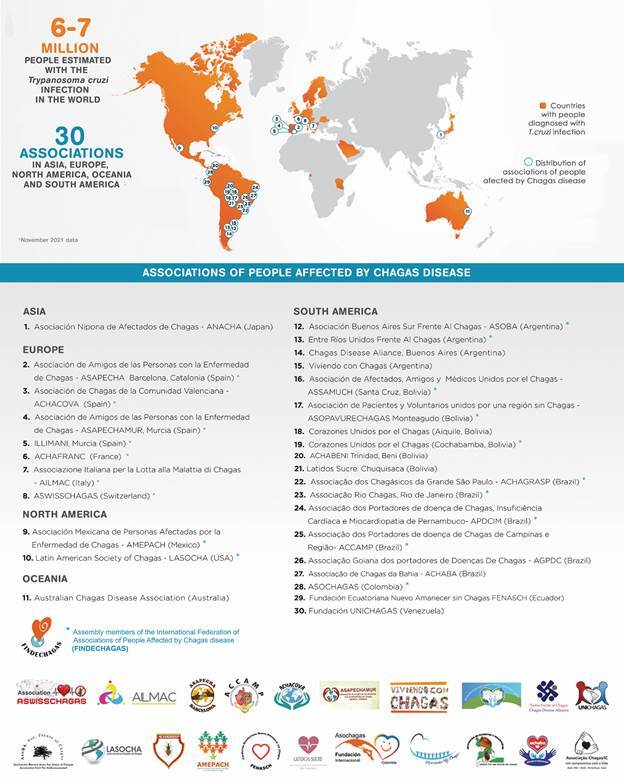
world map of countries with detected people affected by Chagas disease, their associations by continents, and assembly members of FINDECHAGAS, in November 2021.

All thirty associations are non-profit organisations, independently constituted (without dependencies of political parties, religious institutions, commercial interests), run democratically by patients elected at General Assemblies, in accordance with national civil codes. Despite the socioeconomic crisis due to the Coronavirus disease 2019 (COVID-19) pandemic, most of them are currently active or in the process of being legally recognised.

**The world federation creation and its 2009-2019 path**

*Creation of the International Federation of Associations of People Affected by Chagas disease (FINDECHAGAS)* - In April 2009, during the 2nd Catalan Conference on International Health and Tropical Medicine Day, a session called “Chagas disease: 100 years after its discovery” was organised. The first association outside Latin America, ASAPECHA, created the year before, was presented and a subsequent meeting about the CD associative movement in the world was also organised. The participants were the following: Wilson Alves de Oliveira Jr, from the Universidade de Pernambuco/UPE and APDCIM, Recife (Pernambuco); Jordi Gómez i Prat, from the Unit of International Health Vall d’Hebron-Drassanes - PROSICS, Barcelona (Catalonia), Spain and ASAPECHA Barcelona; Paulo Chagastelles Sabrosa, from the National School of Public Health ENSP-Fiocruz, Rio de Janeiro; and Pedro Albajar Viñas, from the WHO CD control programme (Fig. 3). The dream of creating an International Federation of Associations for People Affected by CD, to mark the centenary of the discovery of the disease, that year, was transformed into a plan of immediate execution.

**Fig. 3:**
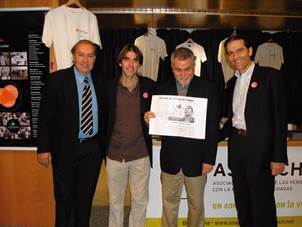
from left to right: Paulo Chagastelles Sabrosa, Jordi Gómez i Prat, Wilson Alves de Oliveira Jr and Pedro Albajar Viñas. Session “Chagas disease: 100 years after its discovery”, in the 2nd Catalan Conference on International Health and Tropical Medicine Day (2009).

Later that year, in October, the dream started to become reality during the 25th Annual Meeting on Applied Chagas Disease Research, in the city of Uberaba, in the Brazilian state of Minas Gerais, with the 1st Meeting of Chagas Disease Patients Associations of the Americas, Europe and Western Pacific (Fig. 4).

**Fig. 4:**
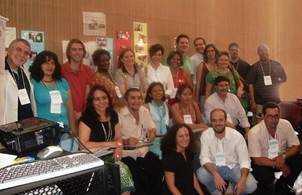
participants of the 1st Meeting of Chagas Disease Patients Associations of the Americas, Europe and Oceania. In the 25th Annual Meeting on Applied Chagas Disease Research, in the city of Uberaba, in the Brazilian state of Minas Gerais (2009).

The objective of this meeting was the creation of an international federation capable of encompassing and strengthening existing associations and encouraging the creation of new civil society organisations, especially in regions where people living with CD are most at risk. This meeting was attended by representatives of Brazilian Society of Tropical Medicine (SBMT), Doctors without Borders (MSF), Fiocruz, Unit of International Health Vall d’Hebron - Drassanes - PROSICS, WHO, and members of various other civil society organisations in Brazil and elsewhere. This first meeting was made possible by the support of the Presidents of the 25th Uberaba meeting, José Rodrigues Coura and João Carlos Pinto Dias. The programme content enabled each association to present their history/origins, mission/objectives, structure and functioning, challenges, and epidemiological data on the disease in their country, together with their thoughts with regard to an international federation.

The event included a presentation of the “History of the disease and action of the civil society”, by Simone Petraglia Kropf, Brazil; “The experience of civil society in the response to the HIV/AIDS epidemics”, by Veriano Terto; “Chagas disease and perceptions”, by Andrea Avaria, Chile; and “The creation of an International Federation”, by the lawyer Pedro Genesca.

Finally, the seed of the future federation was planted and the participants registered their work in the “Uberaba decalogue”, which was read at the close of the 25th Annual Applied Chagas Disease Research Meeting [Supplementary data (I)].

As a consequence of the meeting, the associations emerged around the world began to exchange experiences and form close ties with each other.

As decided at the Uberaba Meeting, the 2nd Meeting of Chagas Patients Associations of the Americas, Europe and West Pacific took place in October 2010, in the city of Olinda, in the Brazilian state of Pernambuco, and was hosted by the APDCIM. This second meeting was promoted by and received support from the DND*i*, MSF, PROCAPE-IAUPE-UPE, and WHO. The event included the first Assembly of the Federation, which approved the first statute and elected ASAPECHA-Barcelona as the association that would take over the presidency in the 2010-2012 two-year period. At the end of the event, those present recorded their work in the “Olinda Charter’’ and collectively signed this document [Supplementary data (II)].

Following the model outlined in Brazil’s Civil Code, during its FINDECHAGAS presidency, ACCAMP, managed to complete the legal registration process in October 2013. The federation was placed on the 1st Official Register of Titles and Documents and Civil Register of Corporations, Campinas-SP, Brazil, under nº 45,232, and on 31/10/2013 was included in the National Company Register (CNPJ) with the nº 19,436,415/0001-30.

*Main decisions and achievements of FINDECHAGAS* - Following its creation, FINDECHAGAS staged four assemblies: the second after its creation in Barcelona/Spain, in 2012; the third in Santa Cruz de La Sierra/Bolivia, in 2014; the fourth in La Plata/Argentina, in 2016; and the fifth in Xalapa/Mexico, in 2018 (Table II).

**TABLE II:** FINDECHAGAS assemblies, place, date and key contents and resolutions

Assembly number	Where	When	Number of participating associations and countries of origin	Key contents and resolutions
First	Olinda, State of Pernambuco (Brazil)	October 2010	Twelve associations from six countries	Approval of the first FINDECHAGAS statute Approval of “Olinda Charter” Supplementary data (II) ASAPECHA-Barcelona was elected president of the federation for the 2010-2012 period
Second	Barcelona, Catalonia (Spain)	April 2012	Fourteen associations, from nine countries	Approval of FINDECHAGAS logo FINDECHAGAS decision to officially register the federation in Brazil. Web-based communication proposed Drew up a fundraising plan Election of 14 April, as the proposed date to celebrate a CD Day worldwide ACCAMP/SP-Brazil, with the support of ACHAGRASP, was elected president of the federation for the 2012-2014 period
Third	Santa Cruz de la Sierra, Department of Santa Cruz (Bolivia)	April 2014	Sixteen associations, from eight countries	FINDECHAGAS decision to become member of the International Alliance of Patients’ Organisations (IAPO) Official proposal of 14 April to become the United Nations World Chagas Disease Day APDCIM/Pernambuco-Brazil was awarded with the title of “Mother Association”, on account of being the oldest. ACCAMP was re-elected president for the 2014-2016 period
Fourth	La Plata, Province of Buenos Aires (Argentina)	April 2016	Seventeen associations, from nine countries	Drawing up a record of the history of the federation Planning joint efforts to strengthen the associations and the federation as a whole. Approval of the “FINDECHAGAS Ten Commandments” Supplementary data (III) AMEPACH was elected president for the 2016-2018 period.
Fifth	Xalapa, State of Veracruz (Mexico)	October 2018	Twenty associations, from eleven countries	Work to seek recognition of 14 April as World Chagas Disease Day by the WHA. Approval of the FINDECHAGAS Facebook and website (www.findechagas.org). AMEPACH was re-elected president for the 2018-2020 period.

In 2020, in the context of the COVID-19 pandemic and travel limitations, FINDECHAGAS incorporates its own video-conference channel (Zoom), with increased regular meetings, improved website (www.findechagas.org) with a repository space for documents, photos and videos. By doing so, it contributed to the “digitalisation” of the federation and its members. In the context of COVID-19, the 6th General Assembly of FINDECHAGAS was successfully organised and developed virtually for the first time. This facilitated a fluent communication between associations and participation of all association members.

**Key contributions and achievements of the associations: innovative experiences**

*Fundamental role of the Chagas disease associations* - Regardless of where the patient lives, associations of people affected by CD have come to play a fundamental role worldwide. They have been working together on citizenship promotion, building a collective identity, sharing knowledge and undertaking political action.^3^ The association movement of CD, as a whole, contributed to the following: (i) individuals affected by the disease are seen as more than just patients, and bring with them geographical and social origins that fueled their epidemiological vulnerability; (ii) a collective identity enables people affected by the disease to feel accepted and create internal mechanisms to transform their experience of suffering into solidarity and mutual support; (iii) CD associations around the world have a variety of different geographic characteristics, but undergo the same struggle; helping to recognise their individual and contextual similarities and improve the well-being of those affected; (iv) both in endemic or non endemic territories the associations have helped to provide accurate information on the disease and its related-problematic.

The need for a comprehensive healthcare approach has contributed to building a solid collective identity and also strengthening active citizenship. An important consideration since the very beginning was how associations and its federation could participate in the design, development and implementation of CD control activities; this was fundamentally based on a multidimensional comprehension and approach.

Below we briefly describe different experiences that, for various reasons, exemplify good practices in incorporating the multiple dimensions mentioned previously of CD into actions. They have greatly contributed to consolidate the CD associative movement with its international federation of associations, its role in healthcare, public health and other fields. In Fig. 5 innovative experiences of the associative movement with their key elements and achievements are put together.

**Fig. 5:**
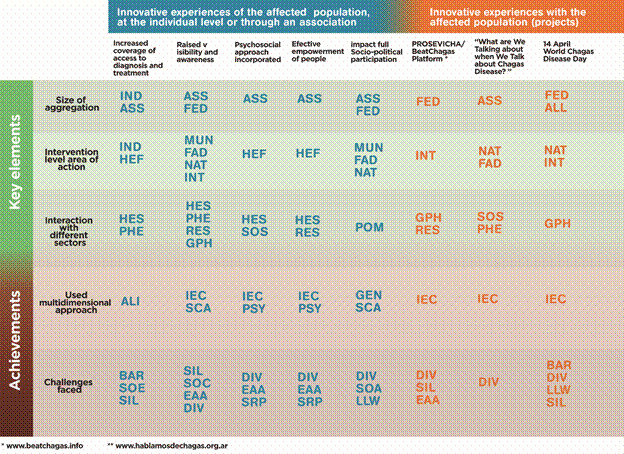
innovative experiences of/with the affected population and associative movement. Key elements and achievements. *IND: individual; ASS: association; FED: federation of associations (FINDECHAGAS); ALL: alliance of associations (IAPO); HEF: health facility; MUN: municipality; FAD: 1st administrative division; NAT: national; INT: international; GLO: global; HES: health system; SOS: social service; PHE: public health; RES: research; POM: policy makers (lows); GPH: global public health; ALI: to deal aspects link to individual acces (place and times of attention, facilitation of transport); GEN: work to deal with gender inequalities (women of childbearing age and congenital transmission…); IEC: information, education and communication approach (unequal information and education, lack of communication about the disease, their rights, out of date information, stereotyped concepts); PSY: work to deal with psychological/personal challenges (fear, shame, isolation…); SCA: inclusion of social and cultural aspects/determinants; BAR: barriers to access to diagnosis and healthcare with followup of a chronic condition; COA: limited continuity/sustainability of actions; DIV: restricted disease visibility and scarce social awareness; EAA: insuficient empowerment and agency; LLW: low interest/lack of social demand/weak political commitment; SIL: silent and silenced disease (low detection rates/epidemiological silence); SOC: social challenges (stigma, exclusion, inequality, discrimination, barriers to access healthcare); SOE: socioeconomic inequalities (related to healthcare, labour rights, social security, life conditions); SRP: insufficient scientific research and product and strategies development.

**Innovative experiences of the affected population and associative movement**

*Increased coverage of access to diagnosis and treatment* - The main aim of the associations has been the collaboration to improve access to diagnosis and treatment. This has been particularly challenging in areas where CD was unknown or just emerging as a public health problem - notably in urban or non-endemic areas. Information and communication activities have been of paramount importance in detection of new cases. They are synergically implemented by associations’ peer educators and health professionals. Sine qua non conditions of success have included: previous training of community agents and trans-sectoral approach with different kinds of health professionals (physicians, nurses, social workers…) coming from different health facilities (primary health centres, hospitals, blood banks…).^16^ ^,^ ^17^ ^,^ ^18^ ^,^ ^19^ ^,^ ^20^ ^,^ ^21^

*Increased visibility and awareness* - Another common objective implemented by all associations has been to increase the visibility of the affected population and raise awareness on CD within the general population. That was especially valuable in order to visualise affected populations in urban areas outside Latin America. The existence, per sé, of an association of people affected by the disease in a municipality sends a strong symbolic message to the authorities on this historically invisible population. In the last few years, several communication experiences and materials have been developed and implemented by associations and are currently available on platforms, such as BeatChagas (https://beatchagas.info/en/home/).^22^ Considering the disease control challenges, it is obvious that additional information and communication work have to be done to increase awareness within the general population.

*Psychosocial approach incorporated* - Since October 2010, a psychosocial approach has been integrated in the biomedical healthcare of CD patients and their relatives in a pioneering way, when the APDCIM joined forces with the PROCAPE-UPE Heart Hospital to open the “Chagas House”.^15^ ^,^ ^23^ It was funded through individual and corporation donations. The idea was to provide office space for the Outpatients Referral Centre on CD, dealing with both coinfections and comorbidities, and an expanded APDCIM head office. This new location has been used to develop activities related to interdisciplinary care, teaching, research, communication (including networks) and outreach services, in addition to the day-to-day work of the association. One activity carried out in the waiting room of the House of Chagas is an innovative approach based on art-therapy to promote learning among patients on solidarity, citizenship and ethics. It is done through the “Art of Loving” and teachings of Chiara Lubich, from the Italian Folklore Movement, with monthly meetings on the “Word of Life” and the “CorArte: Heart with Art” project.^24^

Some of the main achievements of the psychosocial approach to Chagas since the creation of this association have included:

(i) social and political recognition of the organisation as a municipal and state public utility; (ii) unprecedented representation on the University Hospital Oswaldo Cruz/PROCAPE-UPE Research Ethics Committee; (iii) collaboration on policy making to improve public information on the disease and sanitary policies, participating to public hearings in the Chamber of Deputies, in Federal District, Brazil, in 2018, to advocate for mandatory registration of chronic CD cases; (iv) first technical participation of association members in scientific events, such as the session “Voices of those who feel” of the “Brazilian Forum for Neglected Diseases”;^25^ (v) award-winning recognition with the first place at National Successful Experiences in Epidemiology, Prevention and Control of Diseases Show (ExpoEpi, 2017, Federal District/Brazil); (vi) financial sustainability of the association (additionally to donations) achieved by way of a store in the House of Chagas selling new and second-hand goods for the population in need and the reception of goods seised by the Inland Revenue to be sold in the same store (that was possible because they were officially registered and socially recognised).

*Empowerment of people* - The Catalan Expert Chagas Disease Patient programme (CECDP) is a pioneer example of effective empowerment of people affected by CD meant to be shared internationally . Since 2008, ASAPECHA Barcelona has provided support to the community strategies of the espic of the Unit of International Health Drassanes - Vall d’Hebron Hospital/Catalan Institute of Health, in Barcelona. This relation required the development of community intervention strategies to work in a coordinated way.^18^

The CECDP is an initiative that forms part of Catalonia’s Chronic Diseases Programme, implemented by the espic, from Drassanes-Vall d’Hebron International Health Unit, International Health Program of the Catalan Institute of Health - PROSICS. The CECDP aims to increase patient responsibility for their own health and promote self-care. The programme involves nine sessions conducted by an expert patient. The intervention focuses on habits, lifestyle, self-care, knowledge of the disease, perceived health status, self-esteem, participant satisfaction and medical follow-up compliance. Experience and informal knowledge, in combination with technical information, facilitate dialogue and help new patients to accept and adhere to treatment.^26^ In the context of CD, CECDP plays a strong and essential role in imparting knowledge and highlighting the importance of screening to relatives, group members and communities. The CECDP programme has been a successful experience to deal with the migration and health challenge in Catalonia.

One of the main outcomes of the CECDP is the frequent engagement of the participants in the patient associations and their collaborative actions, including updated information sharing and access to diagnosis and treatment facilitation as peer educators.^17^ ^,^ ^18^

*Socio-political participation* - In terms of effective socio-political participation, pioneer and singular examples were led by the Group of Studies on Chagas disease (GEDoCh)/Unicamp and ACCAMP. Over the years, they have greatly helped to produce a socially aware and legally sophisticated approach to strengthening the group, forming an interface with public health authorities and policy makers. At municipal level, ACCAMP has been working systematically, along with the Campinas - SP and Regions Council Chamber, to promote important laws providing protection for individuals affected by CD, such as the Law Nº 15,388 of 22 March 2017 (establishing Neglected Disease Awareness Week in the Municipality of Campinas).^27^ It has also tabled motions at the Ministry of Labour of Brazil asking to abolish the requirement to be tested for CD before starting a new job. And it has also required the Ministry of Health of Brazil to ask for the National Consensus on Diagnosis and Treatment of CD, to become the general policy adopted for providing healthcare of individuals affected by the disease, in the National Health system (SUS).

ACCAMP believes that networking for collective objectives can strengthen social mobilisation and campaigns relating to rights, and improve services and healthcare and social welfare policy. ACCAMP has used its accumulated expertise to help set up new associations, providing information and guidance on the administrative steps that need to be taken to form and officially register as an association.

**Innovative experiences with the affected population (projects)**

*Project to raise awareness and visibility of people affected by CD (PROSEVICHA) and BeatChagas platform* - PROSEVICHA was a clear example of a project developed for and with the affected population. It was a multi-centred multidisciplinary project coordinated by the team of espic of the Unit of International Health Drassanes - Vall d’Hebron Hospital / Catalan Institute of Health, aiming at raising awareness among the general public through social media regarding the situation of people affected by CD (patients, carriers, family, friends, and so forth). It empowered people by giving them the opportunity to gain a better understanding of the complexity of the problem, in particular difficulties related to diagnosis and treatment in a global context. During the first phase, in 2012, two specific lines of action were established: the organisation of the 2nd FINDECHAGAS Assembly - the first in Europe; and the creation of information materials to address the “silence” surrounding CD. A spot with the soccer player Lionel Messi, was designed and developed by PROSEVICHA with the participation of Lionel Messi Foundation and Barça Foundation (https://beatchagas.info/en/resources/spots/spot-messi-1/). To ensure the scientific and ethical principles, the project was based on a qualitative study carried out on the perceptions of those affected by the disease and integrants of the FINDECHAGAS associations, as well as on the professionals working on CD.^22^

In the second phase of the project, in 2013, another information spot on congenital transmission of CD was also created and the launch of a series of songs related to CD was organised at the WHO headquarters, in Geneva, Switzerland.

Since 2015, all created IEC materials and strategies have been shared worldwide through a web platform called BeatChagas. Actually, this Platform is the channel used by the Technical Group 6 on IEC to control CD (TG6IEC-Chagas) of the WHO Chagas disease control programme to communicate and share IEC tools related to the problem of Chagas (www.beatchagas.info).

*What are we talking about when we talk about Chagas? (¿De qué hablamos, cuando hablamos de Chagas?), La Plata, Argentina* - In 2011, at the University of La Plata, Province of Buenos Aires, Argentina, an inter-institutional team of students and professors presented an innovative initiative to apprehend Chagas: “What are we talking about when we talk about Chagas?” (Civil Association let’s talk about Chagas). The group is made up of people with and without Chagas, with different backgrounds: researchers, undergraduate and graduate students, communicators, artists, teachers, health professionals, among others; from different sectors and institutions and organisations, including the Consejo Nacional de Investigaciones Científicas y Técnicas - CONICET, National University of La Plata - UNLP, University of Buenos Aires - UBA, National University Arturo Jauretche - UNAJ, among others (https://hablamosdechagas.org.ar/).

Combining a multitude of voices with an artistic background, the group has aimed: to raise awareness on Chagas in a variety of settings, using conventional and non-conventional educational spaces; to strengthen the role of the IEC in adopting a broad view to this complex problem;^28^ to promote a comprehensive approach of Chagas in different educational and community contexts. This include: (i) awareness raising around Chagas in schools and public spaces; (ii) fostering critical problem reflection with different target audiences and professional teams; (iii) helping training educators, providing fora to exchange information and educational materials (https://hablamosdechagas.org.ar/recursos/). That goes in line with innovative IEC initiatives that have been developed around CD in the recent years.^8^ All of that has been linked with a progressive awareness raising, access to diagnosis and care promotion, speeding up the development of applied research in Argentina.^29^ ^,^ ^30^

The groundbreaking approach developed at La Plata has given materials and foundation to other experiences.

**The 14th April and the World Chagas Disease Day**

One of the resolutions of the second FINDECHAGAS General Assembly, in April 2012, was to organise a CD day worldwide. After considering different possible dates, the federation selected April 14th because Carlos Chagas diagnosed the disease for the first time in a human on that day, specifically in a two years old girl named Berenice, from the Brazilian municipality of Lassance. Following that resolution, an international collaborative process of information dissemination and institutional engagement was initiated. Five years later, in September 2017, Brazil, at the time President of the 142nd session of the WHO Executive Board (EB), submitted a proposal to create a World Chagas Disease Day (WCDD) to be celebrated annually on April 14th.^31^ The proposal was put forward by Brazil, Bolivia, Colombia, Costa Rica, Panama and Spain and notably based on the following facts:

People with CD have been diagnosed in up to 44 countries of five continents (North and Central America, South America, Europe, Africa and Oceania).
The biggest control challenges include early detection and care of people with T. cruzi infection; it is estimated that < 10% of people with CD receive appropriate diagnosis and treatment.
In 2005 CD was already included in the WHO list of Neglected Tropical Diseases. There is a need to raise awareness and address the administrative challenges infected people face.
There is an urge to reduce the discrimination, prejudice and stigma that the affected still face in various aspects of their social, family and working life.
There are numerous challenges regarding the implementation of programs, actions, and comprehensive healthcare protocols for individuals affected by the disease. Those notably include access to timely diagnosis, adequate treatment and followup.

Following the discussions on World Health Days costs and benefits, an analysis was requested to the WHO. In January 2019, during the 144th EB, the report on World Health Days was presented, emphasising the following: international health days generate visibility able to influence policy making, raise general awareness and thereby facilitate fundraising efforts. The submission of the WCDD to the Seventy-second World Health Assembly was endorsed^32^ and, finally, the World Health Assembly, in May 2019, decided to establish World Chagas Disease Day (Fig. 6).^33^

**Fig. 6:**
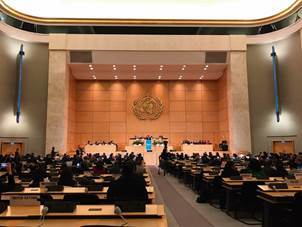
plenary of the Seventy-second World Health Assembly, in Geneva, Switzerland, on 28 May 2019.

This important achievement finalised a nine-year uninterrupted and collaborative process initiated by FINDECHAGAS in 2010, with the unconditional support of IAPO and several actors from different backgrounds, such as: governments, national institutions, nongovernmental organisations and international institutions.

**Lessons learned from the associative movement and a look at future**

Despite all the difficulties and obstacles that people affected by CD have faced since the discovery of the disease, it is clear that patients associations, organised into a federation, have appeared as new key actors at national and international level. They have gained both local and international importance through their participation, knowledge, presence in different events and interaction with many other actors. This has brought about a paradigm shift, whereby the patient is no longer a passive subject in the treatment process, but rather an active protagonist and practitioner of “*patient advocacy”*. This proactive approach, involving critical awareness of one’s own rights and needs, facilitates more effective and appropriate decision-making regarding personal and collective health. Indeed, associations are bridges between civil society and the state, enabling proper participation and follow-up of projects and processes.

In this way, we can clearly visualise the following main future challenges and opportunities. The challenges include:

Consolidation of the associations (based on a functional democratic structure) with the means to achieve empowerment and sustainability, preventing interference from external actors different interests;
Improvement of intercultural intelligence to deal with the internal social diversity of the Federation and with external partners and stakeholders.
Strengthening of the federation unity, with the diversity of associations that compose it in terms of human, historical, cultural, social, economic, geographical characteristics (avoiding understanding unification as homogenisation).
Promotion of partnerships with actors from different backgrounds: governments, private institutions, academia, healthcare system, research centres, among others.
Increasing digital interoperability, internally and with external partners, looking for equitative win-win interactions.

The opportunities include:

1. to continue promoting citizenship in the field of health to ensure that health policies meet the priorities of individuals affected; sticking with slogan drawn up by people with disabilities, “Nothing *for us if not with* us” (calling for co-participation in public health policies formulation);
2. to campaign for multidisciplinary technically competent human healthcare, with comprehensive care for the individual affected, as part of a biopsychosocial approach (participative action research - PAR);
3. to encourage the participation of individuals affected by the disease in scientific and other public events (as the “Voice of one who feels”);
4. to broaden participation of associations and the Federation in social media, using validated IEC tools, thereby improving information and communication on CD (raised visibility and awareness);
5. to effectively empower and educate the patients about their condition, notably, replicating the experiences like the “expert patient” experience (it is untrue that the less the patient knows about their disease, the less they suffer);
6. to break the epidemiological silence; promoting early diagnosis, adequate treatment, and compulsory reporting of acute and chronic cases (increased coverage of diagnosis and treatment and improved notification and surveillance);
7. to keep using digital technology tools, with continued updates and training, to improve day-to-day communication and best practices of work (digital information, education and communication).

Even if pronounced in 1911, the following Carlos Chagas words remain actual. “...There can be no doubt that this health problem presents significant practical difficulties, all of an economic order. However, with the development of employment, agricultural prosperity, repopulation, and the improvement of our local labour force, together with the duties of humanity and of civilisation, the pride of a people and the moral fibre of a nation, we shall certainly not lack the energy needed to confront this problem and, one day, resolve it once and for all”. They indeed evidence the challenges the multidimensionality of CD poses with its interlinked biomedical and psychosocial reality. In that way, it is critical to continue breaking the silenced CD, improve surveillance and reporting, advance in disease control and sustain engagement towards the 2030 agenda.
