# Supplementary material for: How people affected by Chagas disease have struggled with their negligence: history, associative movement and World Chagas Disease Day
Source: Mem Inst Oswaldo Cruz. 2022 Jul 13;117:e220066. doi: 10.1590/0074-02760220066 (PMC9281385; doi:10.1590/0074-02760220066)
Supplement: Supplementary file 1 [file 1678-8060-mioc-117-e220066-s.pdf]

(I)

Uberaba Decalogue. Twentyfifth Annual Applied Chagas Disease Research Meeting and First Meeting of Chagas Disease Patients Associations of the Americas, Europe, and Western Pacific, Uberaba, Minas Gerais, Brazil, in October 2009

Uberaba Decalogue
Uberaba, Minas Gerais, Brazil, October 2009

"By forming an International Federation of People Affected by Chagas Disease, we are addressing this complex problem in an integrated fashion that examines the true circumstances of individuals living with this affliction. Our work is guided by the multifarious and complex nature of this situation and involves solidarity and working together to improve the lives of people affected by Chagas Disease.

We are a group of people that live on a daily basis with Chagas Disease. We are patients, families, and friends affected by Chagas Disease; we are teams that work directly with people affected by it. We aim to include in this network as large a number as possible of people affected by the disease who are well-informed regarding its nature, in order to ensure we have an identity and that our voices are heard. Regardless of place of origin and social circumstances, we declare that we are all individuals with rights and duties in everyday social life, and no effort will be spared to ensure that these rights are upheld.

We realise that information, education and communication are essential tools for building knowledge, countering stigma and indifference, and upholding individual rights. Information, education, and communication regarding the means of transmission are as important as instructions regarding diagnosis, treatment and follow-up, as is understanding of quality of life as a whole.

We advocate the continuation and improvement of prevention initiatives. We understand that prevention is a fundamental component of the struggle to fight the disease. Greater commitment on the part of states and societies will be apparent when they are seen to provide systematic and ongoing vector control, organised blood banks and transplant services, and prevention of oral vertical and other forms of transmission, and to promote more dignified living conditions and improved quality of life among people affected by this disease.

We call for universal access to diagnosis. Physicians and other health workers need to receive adequate training in clinical and laboratory identification of the infection, and to know how to treat the disease professionally and provide appropriate advice for those affected. The availability of tests at health units ensures that everyone has the right to free access to these.

We demand equal universal access to treatment and follow-up during the various phases of the disease. All-round treatment of the patient and of those affected by Chagas Disease involves work by specialists in a multitude of disciplines and social spheres, including lawyers and psychologists. The high cost of technology and bureaucracy should not bar access. The Federation aims to expose this and to collectively seek ways of overcoming such obstacles.

We encourage research and development regarding the best technology. There is a need to prioritize the development of technology, in view of the specific context in which those affected by the disease live. Establishing and strengthening strategic alliances with institutions that develop such technology puts these institutions in touch with the real needs of those affected by Chagas Disease. It is important that these new tools produce fewer side-effects and provide enhanced quality of life.

We unconditionally believe that Chagas Disease does not constitute a death sentence. We need to understand, confront and spread knowledge of the challenges that affect people living with the infection. The cycle of association between poverty and malnutrition and the occurrence of the disease should be broken. We believe that all people have the ability and the right to be employed in dignified work and to receive social welfare, with efforts made to combat malnutrition, prejudice, and other social stigmas. No individual should for any reason undergo diagnostic tests without their consent or have the results revealed and used against them. No individual should be discriminated against on account of having Chagas Disease.

The Federation provides a way of strengthening our joint struggle, providing oversight, and broadening political representation, as a way of upholding fundamental civil rights. United and firm in our commitment, this charter lays out the principles adopted by a global network of people living with Chagas Disease."

(II)

Olinda Charter. First FINDECHAGAS Assembly, Olinda, Pernambuco, Brazil, in October 2010

Olinda Charter
Olinda, Pernambuco, October 2010

"Olinda Manifesto of Uberaba, 19 to 20 October 2009. After analysis of issues raised by Chagas Disease at global, continental, regional and local level involving each association attending the first meeting—the Association of People with Chagas Disease of Greater São Paulo (São Paulo, Brasil), the Association of Friends of People with Chagas Disease (Valencia and Barcelona, Spain), the Association of People with Chagas Disease of Campinas and Environs (Campinas, Brazil), the Association of Chagas Disease Patients with Heart Failure (Recife, Brazil), and the Friends of Chagas Foundation (Venezuela)— on the occasion of the Second Meeting in Olinda, 4 to 6 October 2010, attended by the Chagas Disease Alliance (Argentina), the Association of Friends of People with Chagas Disease (Valencia, Barcelona and Murcia, Spain), The Chagas Disease Patients Foundation for Life (Colombia), the United against Chagas Disease Foundation (Venezuela), Hearts United for Chagas Patients (Cochabamba, Bolivia), the Association for the Campaign against Chagas Disease (Aiquile, Bolivia), the Association of People with Chagas Disease of Greater São Paulo (São Paulo, Brazil), the Association of People with Chagas Disease of Campinas and Environs (Campinas, Brazil), the Association of Chagas Disease Patients with Heart Failure (Recife, Brazil), and the Friends of Chagas Foundation (FUNDACHAGAS, Venezuela), we unanimously ratify the present manifesto declaring that: By forming an International Federation of People Affected by Chagas Disease, we are addressing this complex problem in an integrated fashion that examines the true circumstances of individuals living with this affliction. Our work is guided by the multifarious and complex nature of this situation and involves solidarity and working together to improve the lives of people affected by Chagas Disease. We are a group of people that live on a daily basis with Chagas Disease. We are patients, families, and friends affected by Chagas Disease; we are teams that work directly with people affected by it. We aim to include in this network as large a number as possible of people affected by the disease who are well-informed regarding its nature, in order to ensure we have an identity and that our voices are heard. Regardless of place of origin and social circumstances, we declare that we are all individuals with rights and duties in everyday social life, and no effort will be spared to ensure that these rights are upheld. We realise that information, education and communication are essential tools for building knowledge, countering stigma and indifference, and upholding individual rights. Information, education, and communication regarding the means of transmission are as important as instructions regarding diagnosis, treatment and follow-up, as is understanding of quality of life as a whole. We advocate the continuation and improvement of prevention initiatives. We understand that prevention is a fundamental component of the struggle to fight the disease. Greater commitment on the part of states and societies will be apparent when they are seen to provide systematic and ongoing vector control, organised blood banks and transplant services, and prevention of oral vertical and other forms of transmission, and to promote more dignified living conditions and improved quality of life among people affected by this disease. We call for universal access to diagnosis. Physicians and other health workers need to receive adequate training in clinical and laboratory identification of the infection, and to know how to treat the disease professionally and provide appropriate advice for those affected. The availability of tests at health units ensures that everyone has the right to free access to these. We demand equal universal access to treatment and follow-up during the various phases of the disease. All-round treatment of the patient and of those affected by Chagas Disease involves work by specialists in a multitude of disciplines and social spheres, including lawyers and psychologists. The high cost of technology and bureaucracy should not bar access. The Federation aims to expose this and to collectively seek ways of overcoming such obstacles. We encourage research and development regarding the best technology. There is a need to prioritise the development of technology, in view of the specific context in which those affected by the disease live. Establishing and strengthening strategic alliances with institutions that develop such technology puts these institutions in touch with the real needs of those affected by Chagas Disease. It is important that these new tools produce fewer side-effects and provide enhanced quality of life. We unconditionally believe that Chagas Disease does not constitute a death sentence. We need to understand, confront and spread knowledge of the challenges that affect people living with the infection. The cycle of association between poverty and malnutrition and the occurrence of the disease should be broken. We believe that all people have the ability and the right to be employed in dignified work and to receive social welfare, with efforts made to combat malnutrition, prejudice, and other social stigmas. No individual should for any reason undergo diagnostic tests without their consent or have the results revealed and used against them. No individual should be discriminated against on account of having Chagas Disease. The Federation provides a way of strengthening our joint struggle, providing oversight, and broadening political representation, as a way of upholding fundamental civil rights. United and firm in our commitment, this charter lays out the principles adopted by a global network of people living with Chagas Disease."

Olinda, October 2010

(III)

FINDECHAGAS Ten Commandments. Fourth FINDECHAGAS Assembly, La Plata, Buenos Aires, Argentina, in April 2016

FINDECHAGAS Ten Commandments
La Plata, Buenos Aires, Argentina, April 2016

1. Remember the affiliate organisations and the people affected by the disease in everything you do
2. Commit towards all members behave ethically/transparently
3. Help to uphold the rights of people affected by the disease
4. Respect the aims of the Federation / Respect the Statute
5. Establish effective relationships (healthy relationships)
6. Be loyal to one another
7. Show humanity
8. Value experience of individuals and affiliate organisations
9. Show responsibility / remain true to our principles / Ten Commandments
10. Remain committed to continuing to work together to achieve our goals
